# Three Steps to Improve Management of Noncommunicable Diseases in Humanitarian Crises

**DOI:** 10.1371/journal.pmed.1002180

**Published:** 2016-11-08

**Authors:** Kiran Jobanputra, Philippa Boulle, Bayard Roberts, Pablo Perel

**Affiliations:** 1 Manson Unit, Médecins sans Frontières, London, United Kingdom; 2 Médecins sans Frontières, Geneva, Switzerland; 3 ECOHOST- The Centre for Health and Social Change, London School of Hygiene and Tropical Medicine, London, United Kingdom; 4 Centre for Global Non-Communicable Diseases, London School of Hygiene & Tropical Medicine, London, United Kingdom

## Abstract

Kiran Jobanputra and colleagues argue that better evidence, guidance, and tools are needed to improve the effectiveness and feasibility of noncommunicable disease care in humanitarian settings.

Treatment of noncommunicable diseases (NCDs) is particularly challenging in settings affected by humanitarian crises, where insecurity and damaged health systems reduce access to treatment. While a United Nations (UN) Political Declaration and a World Health Organization (WHO) Global Action Plan recognise the significant contribution of NCDs to global morbidity and mortality [1, 2], the problem of NCDs during emergencies and in humanitarian response has been underrecognised [3]. The evidence base is negligible: a systematic review on the effectiveness of interventions for NCDs in humanitarian settings found just eight studies published over the last 35 years, four of which came from the same refugee camp in Jordan [4]. Humanitarian guidelines (e.g., Sphere) provide scant information on NCDs [5], while leading international NCD guidelines are based on evidence from resource-rich settings and adapted to fit stable, resource-constrained settings [6].

The diversity of the epidemiology of NCDs and health system characteristics in humanitarian crises preclude a simple “one size fits all” approach. The demographics of crisis-affected populations also vary. For example, among Syrian refugees in Jordan, Médecins sans Frontières (MSF) have observed a higher proportion of older people, females, and more frail individuals than among displaced populations in transit within Syria and in neighbouring Lebanon. The Syrian conflict has also displaced populations that have high levels of obesity, diabetes, and cardiovascular disease linked to urbanisation and lifestyle factors [7]. In contrast, in MSF’s experience, displaced Burundians in Tanzania attend clinics most frequently with epilepsy, sickle cell disease, hypertension, and asthma. The specific comorbidities seen in crisis-affected settings (including posttraumatic stress disorder and depression) have a significant impact on presentation and disease course, particularly on hypertension and cardiovascular disease [8]. The quality of health systems in place prior to the crises also vary substantially, as does the ability of the remaining or receiving health system in addressing NCD needs. Similarly, the knowledge of crisis-affected populations on NCD prevention, symptoms, and management will also vary significantly in different contexts.

The UN Interagency Task Force on NCDs (UNIATF) has recently published brief guidance on NCDs in emergencies [9]. Although this is a welcome addition, the principles outlined in the guidance are relatively high level and contain little detail on how to assess NCD needs and implement care in crises. Here, we highlight the key gaps in knowledge and the actions that we believe are required for ensuring NCD care is available to populations in humanitarian crises.

## Improve Understanding of the Needs Related to NCD Care in Crises

There is a lack of informative NCD-related epidemiology in emergency settings, such that in many cases, NCD care provision is based on immediately identified and sometimes presumptive needs, with little data to inform health care priorities or approach. Population-level surveys and digital surveillance systems are starting to provide some understanding of NCD epidemiology amongst displaced populations in the Middle East [10]. However, much more information is needed in general, particularly in rural settings that have been subjected to protracted conflicts. For example, in the conflict-afflicted province of North Kivu in the Democratic Republic of Congo (DRC), health care staff at the MSF-supported referral hospital are managing patients with lifestyle-associated type 2 diabetes as well as patients with malnutrition-related diabetes with very high insulin requirements and early onset of chronic complications [11,12]. The humanitarian health community needs to better understand perceptions and experiences of NCDs in settings that have not historically been affected by these conditions. In a qualitative study in North Kivu, health workers expressed concern about the increasing incidence of diabetes and the difficulties patients would face with self-management, whilst patients and communities described diabetes as a more damning diagnosis than HIV because it caused blindness, disability, and loss of ability to work [13].

## Develop and Evaluate Novel Models of NCD Care in Diverse Humanitarian Contexts

The setup of NCD services in humanitarian settings will vary with resources and geography, preexisting health systems, and the availability and experience of medical staff. For example, MSF’s NCD clinic in Irbid in Jordan is structured along the lines of a European-style chronic care clinic in a primary care centre, with multidisciplinary care, integrated mental health and social work services, and Short Message Service (SMS) text message appointment reminders. By contrast, the MSF diabetes clinic in North Kivu takes the HIV service as its blueprint, in terms of the organisation of patient flow, expert patient counselling, nurse-led care, and use of family members as treatment supporters. Rigorous monitoring and evaluation with cohort analysis will help target interventions to address the weak points in NCD services. Clinical audit is helping us to simplify and streamline these models of care; for example, in a refugee health programme in Jordan, an audit of statin prescribing for prevention of cardiovascular disease found that cholesterol quantification did not increase coverage of appropriate statin prescribing and instead resulted in many patients not having a risk score calculated [14]. The disruption resulting from conflict or humanitarian crises means that treatment support resources (including dietary advice) may need to be adapted in view of changes in financial, food, and physical security experienced by the affected population. High rates of mental trauma may require moving beyond self-care to incorporating family member support for chronic conditions, an approach that we have found to be invaluable in some such settings [13].

## Make Appropriate Tools and Guidance Available

To fill a gap in appropriate clinical guidelines, MSF have developed draft NCD Clinical Guidelines for humanitarian settings, influenced by guidelines from WHO, the National Institute for Health and Care Excellence (NICE) and the European Society for Cardiology (ESC) guidelines (amongst others [6, 15, 16]) but adapting targets, monitoring, and follow-up procedures to account for the specific barriers patients face in such contexts [17]. These guidelines also include a programmatic section, building on lessons learnt from HIV in insecure settings, such as the use of runaway packs (prepacked, patient-held bags of medication), prepositioning of three-month buffer stocks in district hospitals, and decentralisation of services. While we make these guidelines available to all our field teams, we encourage them to make locally appropriate standard operating procedures and health education materials. Furthermore, the MSF guidelines are designed with humanitarian contexts in mind; where national guidelines and tools are available and appropriate, and the setting is sufficiently stable to apply them, MSF teams are encouraged to use these instead. We plan to evaluate the implementation of these guidelines to learn how to improve support for health workers and facilities; in the longer term, prospective research would help further inform guideline development.

## Conclusion

Better evidence, guidance, and tools will improve the effectiveness and feasibility of NCD care in humanitarian settings. Evidence on minimum viable packages for NCD care in different settings will also help inform advocacy efforts, showing donors that good quality care for NCDs is feasible and effective and demonstrating the costs involved. Since NCD care is complex and comorbidity is particularly common in humanitarian settings, health care actors will need to work in a coordinated fashion to ensure relevant services are integrated and patient centred. Partnerships between operational and academic agencies will be critical to ensure that high-quality evidence can be generated to support NCD programming in these difficult settings.

## Abbreviations

ESC: European Society for Cardiology
MSF: Médecins sans Frontières
NCD: noncommunicable disease
NICE: National Institute for Health and Care Excellence
UNIATF: UN Interagency Task Force on NCDs
